# Action of Chloride of Zinc as a Caustic

**Published:** 1860-04

**Authors:** 

**Affiliations:** Surgeon to Hôtel Dieu, at Chartres; Surgeon to Hôtel Dieu, at Chartres


*Action of Chloride of Zinc as a Caustic.* By Salmon and Maunoury, Surgeons to Hôtel Dieu, at Chartres.

The experiments on which the conclusions here given are based, were made in connection with those already furnished our readers, in the last number of the Monthly, p. 188, on the action of caustic potassa. They comprise a series of carefully executed experiments as to the action of chloride of zinc on living and dead tissues.

Chloride of zinc, as compared with caustic potassa, is an agent producing cauterization very slowly. It does not dissolve tissues, but on the other hand, renders them harder and more coriaceous; under the microscope, one can recognize very readily all the anatomical elements of which they are composed. The cauterized surface prevents the ready penetration of the fresh caustic, when it is desired to act at a greater depth; and then it is necessary to remove the eschar, already produced, by caustic potassa, or to cut off the same by the bistoury, or to await its removal, which requires from six to eight days.

It is a caustic which does not spread under the following conditions: 1st. When it is applied on moist or fungous tissues, on a wound, &c. 2d. When the successive layers of the tissue to which it is applied are all of like ready penetrability. But if, under a tissue easily destroyed by the cauterization, an aponeurotic expansion, muscular tissue, &c., be found, it scarcely penetrates these, and spreads through the tissue on which it has been applied, until it doubles, or even triples. the size of the required eschar; hence it cannot, with justice, be said that chloride of zinc destroys tissues like a punch.

It destroys cellular tissue more readily than cutaneous; and the latter more readily than fibrous or muscular tissue, &c. Contrary to the assertion of Girouard, it attacks morbid tissues, such as cancerous growths, with the same facility that it penetrates fungous tissues. If the morbid mass be enveloped with a fibrous covering, the zinc caustic can isolate this mass, but this does not imply the rapidity of its penetration in the morbid tissue when deprived of its envelope. It coagulates the blood even in large vessels, but does not prevent hæmorrhages from following its employment, even when the arteries are only of medium size. Eschars formed by it are soluble in potassa, and this property can frequently be utilized with the view of hastening the termination of cauterizations.—*Gazette Médicale de Paris.*

